# Causal linkage between type 2 diabetes mellitus and inflammatory bowel disease: an integrated Mendelian randomization study and bioinformatics analysis

**DOI:** 10.3389/fendo.2024.1275699

**Published:** 2024-01-19

**Authors:** Xiang Xiao, Xuanyu Wu, Lu Yi, Fengming You, Xueke Li, Chong Xiao

**Affiliations:** ^1^ TCM Regulating Metabolic Diseases Key Laboratory of Sichuan Province, Hospital of Chengdu University of Traditional Chinese Medicine, Chengdu, China; ^2^ Cancer Institute, Chengdu University of Traditional Chinese Medicine, Chengdu, China

**Keywords:** type 2 diabetes mellitus, colorectal cancer, inflammatory bowel disease, Crohn’s disease, ulcerative colitis, single nucleotide polymorphisms, bidirectional Mendelian randomization

## Abstract

**Background:**

Observational studies have indicated associations between type 2 diabetes mellitus (T2DM) and both colorectal cancer (CRC) and inflammatory bowel disease (IBD). However, the underlying causality and biological mechanisms between these associations remains unclear.

**Methods:**

We conducted a bidirectional Mendelian randomization (MR) analysis employing summary statistics from genome-wide association studies involving European individuals. The inverse variance weighting (IVW) method was the primary method used to assess causality. Additionally, we applied MR Egger, Weighted median, Simple mode, and Weighted mode to evaluate the robustness of the results. Outliers were identified and eliminated using the MR-PRESSO, while the MR-Egger intercept was used to assess the horizontal pleiotropic effects of single nucleotide polymorphisms (SNPs). The heterogeneity was evaluated using the Cochrane *Q* test, and sensitivity analysis was performed using leave-one-out method. The *F* statistic was calculated to evaluate weak instrumental variable bias. Finally, a pilot bioinformatics analysis was conducted to explore the underlying biological mechanisms between T2DM and IBD/UC.

**Results:**

The IVW results demonstrated that T2DM significantly reduced risks of IBD (*OR*=0.885, 95% *CI*: 0.818–0.958, *P*=0.002) and ulcerative colitis (UC) (*OR*=0.887, 95% *CI*: 0.812–0.968, *P*=0.007). Although the 95% *CI*s of MR Egger, Weighted median, Simple mode, and Weighted mode were broad, the majority of their estimates were consistent with the direction of IVW. Despite significant heterogeneity among SNPs, no horizontal pleiotropy was observed. The leave-one-out analysis showed that the causality remained consistent after each SNP was removed, underscoring the reliability of the results. Reverse MR analysis indicated that genetic susceptibility to both CRC and IBD had no significant effect on the relative risk of T2DM. Ten hub genes were identified, which mainly enriched in pathways including maturity onset diabetes of the young, thyroid cancer, gastric acid secretion, longevity regulating pathway, melanogenesis, and pancreatic secretion.

**Conclusion:**

The presence of T2DM does not increase the risk of CRC or IBD. Moreover, T2DM might reduce risk of IBD, including UC. Conversely, the occurrence of CRC or IBD does not influence the risk of T2DM. The association between T2DM and IBD/UC may be related to the changes in multiple metabolic pathways and CTLA-4-mediated immune response.

## Introduction

Colorectal cancer (CRC) manifests as a malignant tumor originating from the colorectal mucosal epithelium. Currently, CRC is the third most common malignancy worldwide in terms of morbidity and the second leading cause of mortality, imposing a substantial social and economic burden (1). CRC is influenced by numerous risk factors and exhibits robust associations with both genetic and environmental factors. Emerging evidence underscores inflammatory bowel disease (IBD) as a key precursor to CRC (2). IBD primarily encompasses two forms: ulcerative colitis (UC) and Crohn’s disease (CD). Prolonged inflammatory processes can result in the abnormal proliferation of colonic mucosa, thereby elevating the risk of CRC. Although the effect of environmental factors on CRC and IBD remains unclear, certain exposures, such as smoking, alcohol consumption, obesity, and type 2 diabetes mellitus (T2DM), have been considered potentially pivotal contributors (3).

T2DM is a glucose metabolism disorder characterized by relative insulin deficiency, ranking among the most prevalent chronic metabolic disorders. The Global Burden of Disease Study projected a global diabetic population of 529 million in 2021, with 96% attributed to T2DM. Furthermore, estimates suggest that the number of individuals with diabetes globally will rise to 1.31 billion by 2050 (4). Prolonged abnormal glucose metabolism associated with T2DM can induce chronic inflammation in the body through mechanisms such as oxidative stress and immune dysregulation.

Observational studies have indicated a link between T2DM and both CRC and IBD. T2DM is considered to influence the occurrence, development, treatment, and prognosis of CRC and IBD; however, establishing causality remains elusive. The previously observed associations could be influenced by confounding factors, such as obesity, that are associated with them. Some perspectives suggest that CRC and IBD increase the risk of T2DM (5, 6). This supposition holds merit, given that the gut plays a critical role in glucose homeostasis regulation, hinting at shared pathogenesis between IBD and T2DM (7). Furthermore, drugs used in IBD treatment can influence glucose metabolism (8).

Mendelian randomization (MR) research employs the instrumental variable method as its core principle, with single nucleotide polymorphisms (SNPs) serving as instrumental variables (IVs). SNPs, assigned randomly at conception, remain unaffected by confounding factors and reverse causation, thus ensuring the reliability of MR in causal inference studies (9). Therefore, we aimed to elucidate the potential dual causality between T2DM and both CRC and IBD by conducting a bidirectional, two-sample MR investigation. MR analysis was first performed using SNPs with established influence on T2DM as IVs to provide indirect evidence of a causal association between T2DM and CRC/IBD risk. Subsequently, MR analysis was performed using SNPs known to affect CRC/IBD as IVs to provide indirect evidence of their reverse causal association. Finally, we conducted protein-protein interaction (PPI) network analysis and enrichment analysis to investigate the potential biological mechanism between the diseases.

## Materials and methods

### Bidirectional two-sample Mendelian randomization study

#### Study design

**Figure 1:**
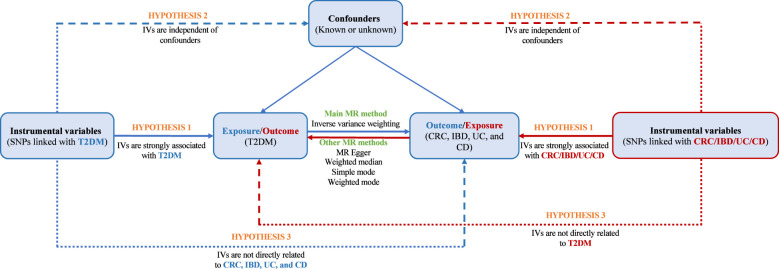
Flow chart of the bidirectional MR study.

In this study, we used pooled data from published studies and open-source genome-wide association studies (GWAS) to evaluate the causality between T2DM and both CRC and IBD. MR research must satisfy the following three core assumptions: relevance, independence, and exclusivity. Hypothesis 1: IVs must exhibit a strong association with exposure factors; Hypothesis 2: IVs cannot be associated with any confounding factors; Hypothesis 3: IVs can only affect outcomes through exposure factors (**Figure 1**). The data used in this study were extracted from published studies and databases, eliminating the need for additional ethical approval and informed consent.

#### GWAS datasets for T2DM and CRC/IBD

We only used GWAS data derived from European populations to avoid bias caused by differences in allele frequencies and linkage disequilibrium between European and non-European populations (10). The most recent GWAS summary data with the largest sample size were incorporated for IVs screening. **Table 1** shows the GWAS datasets employed in this study. Summary statistics for T2DM were obtained from the Diabetes Meta-Analysis of Trans-Ethnic association studies (DIAMANTE) and recent data from the Type 2 Diabetes Knowledge Portal (T2DKP) (11, 12). The DIAMANTE included 74,124 T2DM patients and 824,006 healthy controls, and T2DKP provided data on 545 significant SNPs (*P*<5×10^-8^) from European T2DM patients. For CRC, summary statistics were acquired from the IEU Open GWAS project database and recent GWAS meta-analyses. Specifically, the ieu-b-4965 dataset, sourced from the IEU Open GWAS project database, comprised 5,657 individuals diagnosed with CRC, and 372,016 healthy controls were obtained. Furthermore, we acquired aggregated GWAS data from Jiang et al., encompassing 636 CRC patients and 455,640 healthy controls (13). Additionally, the recently published meta-analysis of Ceres et al. was incorporated, involving 78,473 CRC patients and 107,143 healthy controls (14). Summary statistics on IBD, including UC and CD, were sourced from the latest GWAS results from FinnGen (DF9). Specifically, the data of IBD was obtained from 377,277 individuals (7,625 cases and 369,652 controls); Data of UC was obtained from 376,564 individuals (5,034 cases and 371,530 controls); Data of CD was obtained from 361,934 individuals (2,007 cases and 359,927 controls).

**Table 1:** GWAS datasets for T2DM and CRC/IBD.

Traits	*N* case	*N* control	Population	Date	Data accession address
T2DM	74,124	824,006	European	2022	PMID: 35551307
	–	–	European	2023	https://t2d.hugeamp.org/
CRC	5,657	372,016	European	2021	https://gwas.mrcieu.ac.uk/
	636	455,640	European	2021	PMID:34737426
	78,473	107,143	European	2023	PMID:36539618
IBD	7,625	369,652	European	2023	https://www.finngen.fi/fi
UC	5,034	371,530	European	2023	https://www.finngen.fi/fi
CD	2,007	359,927	European	2023	https://www.finngen.fi/fi

#### Selection of IVs

Referring to a previous study, IVs were screened through the following steps (1): Identification of SNPs closely related to exposure factors with a significance standard threshold of *P*<5×10^-8^; (2) Elimination of SNPs in linkage disequilibrium with a standard of r^2 = ^0.001 and kb=10,000; (3) Calculation of the *F* statistic (β^2^/SE^2^) to assess weak IV bias. An *F* value below 10 indicates a weak IV bias, which might lead to an underestimation of statistical power (15); (4) Exclusion of IVs related to confounding such as obesity using PhenoScanner (http://www.phenoscanner.medschl.cam.ac.uk/), which provided SNP phenotyping information was employed. In instances where a disease corresponds to multiple sources of GWAS summary data, IVs were initially screened individually according to the aforementioned steps, followed by the removal of duplicate SNPs to yield the final IVs.

#### Statistical analysis

IVs were initially screened and coordinated using the TwoSampleMR software package (version 0.5.7). Subsequently, outliers were detected and removed using the MR-PRESSO package (version 1.0). In order to estimate the causal effect, the primary analysis method used was inverse variance weighting (IVW). IVW is an extension of the Wald ratio estimator that is based on the principle of meta-analysis. Additionally, MR Egger, Weighted median, Simple mode, and Weighted mode were employed to ensure result consistency. As the outcome was a dichotomous variable, the odds ratio (*OR*) was used to assess the potential causality between exposure and outcome. Given that traditional IVW methods might be influenced by ineffective instrument bias or pleiotropic effects, a leave-one-out sensitivity analysis was performed to test the validity of IVW results. Furthermore, the robustness of the results was evaluated; the Cochrane *Q* test assessed heterogeneity, and the MR-Egger intercept test evaluated horizontal pleiotropic effects. All statistical tests were two-tailed, and all statistical analyses were performed using RStudio (version 2023.03.0 + 386). Statistical significance was set at *P*<0.05.

### Pilot bioinformatics analysis

#### Data collection

First, the mapped genes of the SNPs used in the MR analysis were collected from the GWAS catalog database (https://www.ebi.ac.uk/gwas/). Subsequently, the GeneCards database (https://www.genecards.org/) was employed to obtain genes related to inflammation and glucose metabolism.

#### Protein-protein interaction network analysis and function enrichment analysis

PPI network analysis of overlaps of mapped genes of SNPs, inflammation-related genes and glucose metabolism-related genes was conducted using STRING database (https://cn.string-db.org/). The Cytoscape software was employed to identify the top 10 hub genes by calculating degree of each gene (16). In addition, GO and KEGG enrichment analyses for the hub genes were conducted using the Bioinformatics online platform (http://www.bioinformatics.com.cn/).

## Results

### Selection of IVs and assessment of weak instrument bias

From the above eight GWAS summary data, there were 1,798, 322, 5,023, 8, 897, 5,201, 4,202, 553 SNPs in linkage disequilibrium were eliminated from original GWAS data. According to the outlined criteria, we examined 158, 20, 47, 34, and 10 SNPs as IVs for T2DM, CRC, IBD, UC, and CD, respectively (**Supplementary Tables 1**–**5**). Notably, in this MR study, we detected and eliminated outliers using MR-PRESSO, resulting in a reduced actual count of SNPs used for MR analysis compared to the initial number. The *F* statistics for the included SNPs in the study exceeded 10, indicating the absence of weak IV bias and ensuring the reliability of the research results (**Supplementary Table 6**). Four SNPs, rs10146997, rs10938397, rs12970134, and rs2112347, which were related to obesity were excluded by searching PhenoScanner.

### Causal association between T2DM and CRC/IBD

**Figure 2:**
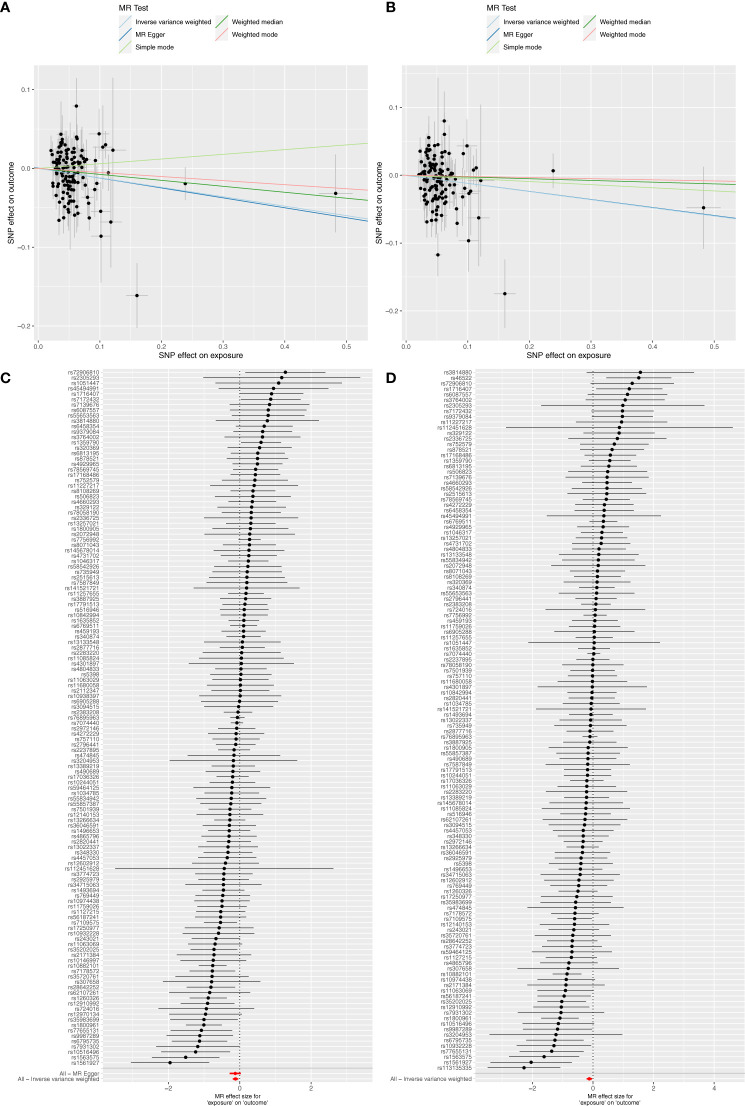
Scatter plots and forest plots illustrate the causal effects of T2DM on IBD and UC. **(A)** Scatter plots showed that T2DM significantly reduced the risk of IBD; **(B)** Scatter plots showed that T2DM significantly reduced the risk of UC; **(C)** Forest plot of MR analysis results for T2DM on IBD; **(D)** Forest plot of MR analysis results for T2DM on UC.

The MR results indicated causal associations between T2DM and both IBD and UC, but no such associations were found regarding CRC and CD. Conversely, reverse MR findings revealed that CRC and IBD did not exert any causal effect on T2DM (**Table 2**). Specifically, T2DM significantly lowered the risk of IBD (*OR*=0.885, 95% *CI*: 0.818–0.958, *P*=0.002) and UC (*OR*=0.887, 95% *CI*: 0.812–0.968, *P*=0.007). Although the 95% *CI*s for the remaining four methods (MR Egger, Weighted median, Simple mode, and Weighted mode) were wide, their estimated values largely aligned with the IVW direction (**Figure 2**; **Supplementary Table 7**). **Supplementary Figure 1** provides scatterplots of the residual causality between T2DM and CRC/IBD.

**Table 2:** IVW results of MR analyses of the dual causal effects between T2DM and CRC/IBD.

Exposure	Outcome	Nsnp	*β*	SE	*OR(95%CI)*	*Pval*
T2DM	CRC	153	0.001	0.001	1.001(0.999~1.002)	0.373
T2DM	IBD	126	-0.122	0.04	0.885(0.818~0.958)	0.002
T2DM	UC	127	-0.120	0.045	0.887(0.812~0.968)	0.007
T2DM	CD	133	-0.108	0.063	0.897(0.793~1.016)	0.087
CRC	T2DM	10	-0.046	0.067	0.955(0.837~1.089)	0.492
IBD	T2DM	7	0.000	0.023	1.000(0.955~1.047)	0.999
UC	T2DM	7	0.007	0.023	1.007(0.963~1.053)	0.749
CD	T2DM	1	0.007	0.030	1.007(0.95~1.068)	0.815

### Sensitivity analysis

**Figure 3:**
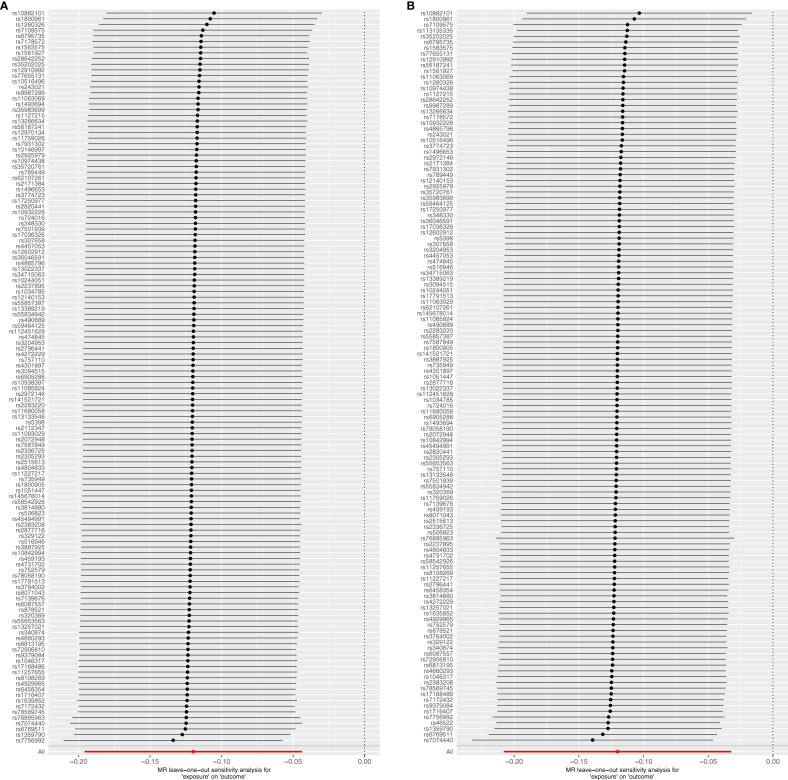
Forest plots of leave-one-out sensitivity analysis indicate the validity of IVW results. **(A)** T2DM on IBD; **(B)** T2DM on UC.

**Figure 4:**
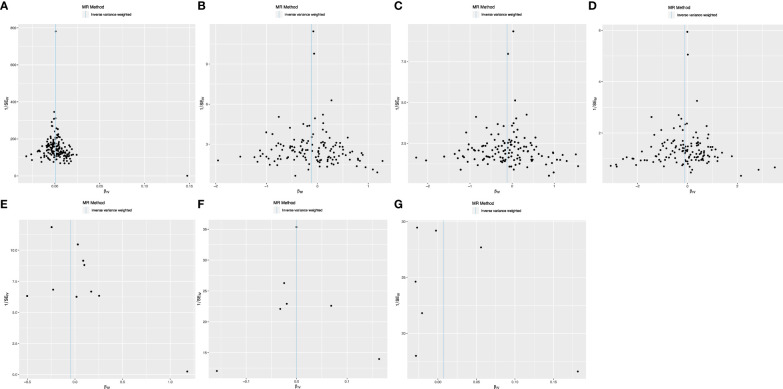
Funnel plots demonstrate the absence of pleiotropy among the included SNPs. **(A)** T2DM on CRC; **(B)** T2DM on IBD; **(C)** T2DM on UC; **(D)** T2DM on CD; **(E)** CRC on T2DM; **(F)** IBD on T2DM; **(G)** UC on T2DM.

The Cochrane *Q* test highlighted notable heterogeneity across the SNPs in most MR analyses (*P*<0.05), except those encompassing SNPs in the causal association between IBD and T2DM (*P*>0.05). Such heterogeneity is acceptable in MR studies. Additionally, the MR-Egger intercept test indicates the absence of horizontal pleiotropic effects among the included SNPs (*P*>0.05; **Table 3**). Subsequently, a leave-one-out sensitivity analysis was conducted to verify the effect of each SNP on the overall causality. The results indicated that the causality remained significantly consistent even when individual SNPs were systematically removed, suggesting that no single SNP could account for the estimated causal effects (**Figure 3**; **Supplementary Figure 2**). The funnel plot exhibited no signs of horizontal pleiotropy in this study (**Figure 4**), corroborating the reliability of the results.

**Table 3:** Results of sensitivity analysis.

Exposure	Outcome	nSNP	*Pval* of Cochrane *Q* test	MR-Egger intercept	*Pval* of Pleiotropy
T2DM	CRC	153	0.002	<0.001	0.787
T2DM	IBD	126	<0.001	0.001	0.906
T2DM	UC	127	<0.001	<0.001	0.984
T2DM	CD	133	0.027	-0.003	0.728
CRC	T2DM	10	0.003	0.011	0.355
IBD	T2DM	7	0.054	0.002	0.870
UC	T2DM	7	0.045	0.007	0.609
CD	T2DM	1	*NA*	*NA*	*NA*

### Potential biological mechanisms between T2DM and IBD/UC

**Figure 5:**
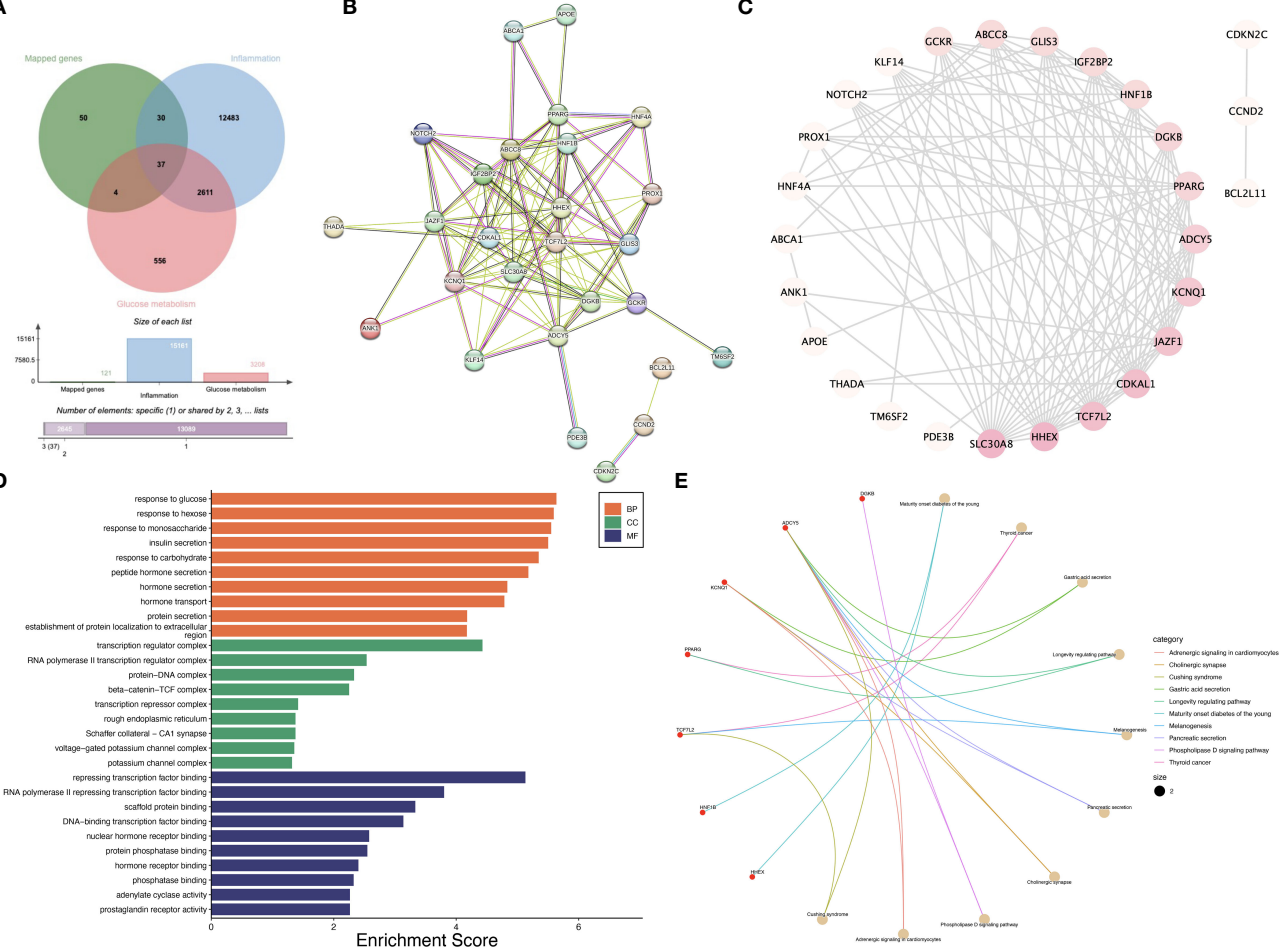
Pilot bioinformatics analysis initially explored the potential biological mechanisms between T2DM and IBD/UC. **(A)** Venn diagram showed that there were 37 overlapping genes; **(B)** PPI network showed that 27 genes interacted with each other; **(C)** The top 10 genes were screened according to Degree value; **(D)** GO enrichment analysis of the ten hub genes; **(E)** KEGG enrichment analysis of the ten hub genes.

We obtained 121 mapped genes of SNPs used in the MR analysis, 15,161 inflammation-related genes, and 3,208 glucose metabolism-related genes. There are 37 overlapping genes between the above genes (**Figure 5A**). The PPI network analysis screened out 27 interacting genes (**Figure 5B**). Subsequently, the top ten hub genes with the highest Degree were identified, which were *SLC30A8, TCF7L2, HHEX, CDKAL1, JAZF1, KCNQ1, ADCY5, PPARG, DGKB*, and *HNF1B* (**Figure 5C**). GO analysis showed that hub genes were mainly enriched in response to glucose, response to hexose, response to monosaccharide, insulin secretion, transcription regulator complex, RNA polymerase II transcription regulator complex, protein-DNA complex, transcription regulator complex, RNA polymerase II transcription regulator complex, protein-DNA complex, beta-catenin-TCF complex, repressing transcription factor binding, RNA polymerase II repressing transcription factor binding, scaffold protein binding, DNA-binding transcription factor binding, and nuclear hormone receptor binding (**Figure 5D**). KEGG analysis showed that hub genes were mainly enriched in pathways such as Maturity onset diabetes of the young, Thyroid cancer, Gastric acid secretion, Longevity regulating pathway, Melanogenesis, Pancreatic secretion, Cholinergic synapse, and Phospholipase D signaling pathway (**Figure 5E**).

## Discussion

In this study, we systematically assessed the causality between T2DM and both CRC and IBD using bidirectional MR analysis. The results demonstrated that the genetic susceptibility of T2DM was linked to a reduced risk of IBD and UC. A series of sensitivity analyses further validated this negative correlation. Conversely, reverse MR analysis indicated that the genetic predisposition to CRC/IBD was not significantly associated with the susceptibility to T2DM. To the best of our knowledge, this is the first study to conduct MR analysis using the latest large-scale GWAS summary statistics to explore the bidirectional causality between T2DM and CRC/IBD, thereby enhancing and advancing prior research.

The genetic susceptibility of T2DM was linked to a reduced risk of IBD, including UC. This finding was not found in earlier observational studies. The underlying mechanism by which T2DM lowers the risk of IBD, particularly UC, remains unclear. The results of this study suggested that rs2796441 and rs3887925 could potentially mediate this association. Positioned near *TLE1* on chromosome 9, rs2796441 can induce *TLE1* intronic mutations. *TLE1* plays a key role in suppressing glucagon in T2DM (17). It can negatively regulate the inflammatory response mediated by the NOD2/NF-κB pathway, which contributes to the onset of IBD (18, 19). Located near *ST6GAL1* on chromosome 3, rs3887925 can induce *ST6GAL1* intronic mutations. Inhibition of *ST6GAL1* downregulates NF-κB and simultaneously reduces the production of pro-inflammatory factors, consequently exerting an inhibitory effect on UC (20). This evidence provides partial insights into the mechanism underlying the potential of T2DM to reduce the risk of IBD, including UC.

A pilot bioinformatics analysis was conducted to explore the underlying biological mechanisms between T2DM and IBD, including UC. There were ten genes, *SLC30A8*, *TCF7L2*, *HHEX*, *CDKAL1*, *JAZF1*, *KCNQ1*, *ADCY5*, *PPARG*, *DGKB*, and *HNF1B* were identified as the hub genes between T2DM and IBD, including UC. A large number of studies have confirmed that *TCF7L2* and *CDKAL1* are susceptibility genes for IBD (21–24). *KCNQ1* exists on the surface of colon cells and controls potassium channels. The *KCNQ1/KCNE3* pathway is active in UC and leads to Na^+^ absorption defects, the primary pathophysiological mechanism causing UC diarrhea (25). *PPARG* is decreased in UC mice, and plays an important role in modulating the M1/M2 polarization of macrophages, which has been proven to take part in the development of UC (26, 27).

Although the genetic susceptibility of T2DM is associated with a diminished risk of IBD, including UC, the potential adverse effects of T2DM on IBD should not be disregarded. First, T2DM treatment could constitute a risk factor for IBD. For instance, the hazard ratio (*HR*) for IBD development in T2DM patients treated with dipeptidyl peptidase-4 inhibitors may be as high as 2.9 (28). In addition, T2DM can influence the clinical treatment outcomes of IBD. A recent large-sample meta-analysis revealed that IBD patients with coexisting T2DM exhibited significantly increased risks of pulmonary (*OR*=1.72) and urinary tract (*OR*=1.93) infections, and they were highly susceptible to sepsis (*OR*=1.56) (29). Furthermore, T2DM can exacerbate IBD. Compared with patients solely affected by IBD, those with concomitant T2DM displayed elevated C-reactive protein levels, erythrocyte sedimentation rates, and eosinophil and monocyte counts, along with reduced albumin levels. This subgroup often required higher doses and prolonged courses of 5-aminosalicylic acid, narcotics, and antibiotics (30).

Previous studies have yielded conflicting results concerning the association between T2DM and CRC. Data from Sweden suggested that T2DM diagnosis before the age of 50 correlated with a 1.9-fold increased risk of CRC before the same age, while another meta-analysis indicated a 1.3-fold risk increase (31, 32). However, a case-control study conducted in the United States contradicted this finding (33). The MR analysis results indicated a lack of genetic-level causality between T2DM and CRC.

Crosstalk between T2DM and CRC is intriguing. Previous studies have elucidated that T2DM can induce or aggravate CRC through epigenetic pathways. The hyperglycemic state of T2DM promotes the production of ROS, RNS, and AGEs within cells. This, in turn, triggers intestinal barrier dysfunction and dysbiosis, increases the permeability of bacteria and toxins, and amplifies TLR signaling in intestinal epithelial cells and associated immune cells. Consequently, systemic inflammation ensues, resulting in an imbalance in the immunological response, diminished immune surveillance functionality, cell transformation, and promotion of angiogenesis, all of which contribute to the onset of CRC (34). Moreover, hyperglycemia can accelerate CRC progression by activating BMP4 signaling (35). In addition, excessive adipose tissue accumulation disrupts the balance between leptin and adiponectin, thereby promoting CRC proliferation and invasion (36). Transcriptomic analyses have revealed significant alterations in cytokine and metabolism-related pathways in tumor-infiltrating CD8^+^ T cells among individuals with both T2DM and CRC compared with those with CRC alone (37). Furthermore, T2DM significantly affects the prognosis of CRC, with one prospective cohort study revealing a substantial increase in CRC mortality (*HR*=2.58) (38). A recent meta-analysis established that metformin, the first-line drug for T2DM, can prevent adenoma occurrence (*RR*=0.77) and prolong overall survival in CRC patients (*HR*=0.6) (39). Preclinical studies have also verified that metformin can inhibit CRC growth by inhibiting the TGF-β/PI3K/AKT signaling pathway (40). Moreover, T2DM increases the risk of premalignant lesions. Epidemiological data indicate that hyperinsulinemia in T2DM increases the risk of colorectal adenoma (*OR*=1.8), which is a critical precancerous lesion of CRC (41).

Although T2DM is primarily a metabolic disease, many studies have focused on the role of immune disorders in the condition. A recent study reported the development of T2DM in melanoma patients receiving anti-CTLA-4 immunotherapy for a long time (42). Additionally, downregulation of CTLA-4 expression plays a catalytic role in various autoimmune diseases, including IBD (43). CTLA-4 shows the same expression trend in T2DM and IBD, suggesting we can explore the common biological mechanism between the two diseases through CTLA-4. Furthermore, previous studies have confirmed that CTLA-4 is crucial in down-regulating T-cell immune function (44, 45). CTLA-4 is upregulated in various cancers, including CRC, to promote disease progression (46). Anti-CTLA-4 therapy has become an essential treatment for a variety of tumors, including CRC (47). Based on the clinical study results of CheckMate142, nivolumab ± ipilimumab is now recommended for treating all lines of advanced colorectal cancer with MSI-H/dMMR (48). In addition, the long-term follow-up of the GERCOR NIPICOL Phase II study found that 1-year, 2-year, and 3-year progression-free survival of 75.4%, 70.0%, and 70.0% for MSI-H/dMMR metastatic CRC treated with dual immunotherapy with nivolumab ± ipilimumab was possible (49). Besides, capecitabine, a key chemotherapy drug for CRC, can also reduce the expression of CTLA-4 in CRC tissue samples and cell lines (50). Although the mechanism by which capecitabine interferes with the expression of CTLA-4 in CRC tissues and cells is still unknown, this evidence is sufficient to reflect the critical role of CTLA-4 in the development of CRC. In summary, there is considerable evidence that CTLA-4 plays a vital role in the occurrence, development, and treatment of T2DM, IBD, and CRC. Furthermore, this MR analysis also found a significant association between T2DM and IBD. Hence, it is crucial to actively explore the role of CTLA-4 in this association in future studies.

Reverse MR analysis revealed that the genetic susceptibility of CRC/IBD was not significantly associated with the risk of T2DM, diverging from the findings of previous observational investigations. In a nationwide cohort study in Denmark, a standardized incidence rate (*SIR*) of 1.54 was established for T2DM among IBD patients, with a SIR of 1.54 for UC and 1.57 for CD (5). Concurrently, in a population study in South Korea, IBD patients had an increased risk of T2DM (*HR*=1.135), particularly in CD patients (risk elevated by 1.677 times) (51). In an earlier analysis of data from the UK Clinical Practice Research Datalink, UC patients exhibited a signifi cantly increased risk of T2DM (*HR*=1.26) (52). Similarly, the probability of T2DM in CRC patients was significantly increased (*IHR*=1.21) (6). The associations established in previous observational studies were not attributable to genetic inheritance but rather could be attributed to confounding factors or epigenetic modifications. For instance, IBD treatment drugs (aminosalicylates, corticosteroids, thiopurines, and biologics) can influence blood glucose control, leading to glucose intolerance and diabetes (8). Recent research highlights that RAGE, a key molecule in IBD pathogenesis, can potentially contribute to the onset of T2DM through alterations in intestinal permeability (53).

This study has limitations that should be acknowledged. Firstly, the MR analysis only utilized SNPs from European individuals, which may limit the generalizability of the findings to other populations. Therefore, it is necessary to conduct further MR studies in diverse populations. Additionally, it is important to note that MR can only elucidate causal associations from a genetic standpoint, and not those influenced by acquired factors. Consequently, more comprehensive molecular mechanism studies are warranted to uncover the potential impacts of T2DM on CRC and IBD. Conversely, this study possesses several notable strengths, including the utilization of the most recent and extensive GWAS data for the MR analysis. Simultaneously, the incorporation of a substantial sample population mitigated the bias arising from population stratification to the greatest extent feasible.

## Conclusion

Many observational studies have shown that T2DM is associated with IBD and CRC. However, limited research has proposed causal associations and biological mechanisms between them. Therefore, we performed this MR analysis combined with pilot bioinformatics analysis study. The findings of this study indicate that T2DM does not increase the risk of CRC/IBD. On the contrary, T2DM could potentially decrease the risk of IBD, particularly UC, to a certain extent. The association between T2DM and IBD/UC may be related to the changes in multiple metabolic pathways and CTLA-4-mediated immune response. Nevertheless, the intricate interplay between T2DM and CRC/IBD at the epigenetic level necessitates further research. A comprehensive investigation into their interactive mechanism holds vital implications for both T2DM and CRC, as well as its precancerous inflammatory lesions.

## Data availability statement

The original contributions presented in the study are included in the article/**Supplementary Material**. Further inquiries can be directed to the corresponding authors.

## Ethics statement

Ethical approval was not required for the study involving humans in accordance with the local legislation and institutional requirements. Written informed consent to participate in this study was not required from the participants or the participants’ legal guardians/next of kin in accordance with the national legislation and the institutional requirements.

## Author contributions

XX: Conceptualization, Software, Writing – original draft. XW: Conceptualization, Software, Writing – original draft. LY: Formal Analysis, Writing – original draft. FY: Supervision, Writing – review & editing. XL: Supervision, Writing – review & editing. CX: Funding acquisition, Supervision, Writing – review & editing.

## Supplementary material

Supplementary Table 1 SNPs employed with T2DM as exposure factor.

Supplementary Table 2 SNPs employed with CRC as exposure factor.

Supplementary Table 3 SNPs employed with IBD as exposure factor.

Supplementary Table 4 SNPs employed with UC as exposure factor.

Supplementary Table 5 SNPs employed with CD as exposure factor.

Supplementary Table 6 F statistic of SNPs employed in MR analysis.

Supplementary Table 7 MR results of MR Egger, Weighted median, Simple mode, and Weighted mode.

Supplementary Figure 1 Scatterplots of the causality between T2DM and CRC/IBD.

Supplementary Figure 2 Forest plots of the results of leave-one-out.
