# Supplementary figures and images for: Causal linkage between type 2 diabetes mellitus and inflammatory bowel disease: an integrated Mendelian randomization study and bioinformatics analysis
Source: Front Endocrinol (Lausanne). 2024 Jan 19;15:1275699. doi: 10.3389/fendo.2024.1275699 (PMC10836595; doi:10.3389/fendo.2024.1275699)

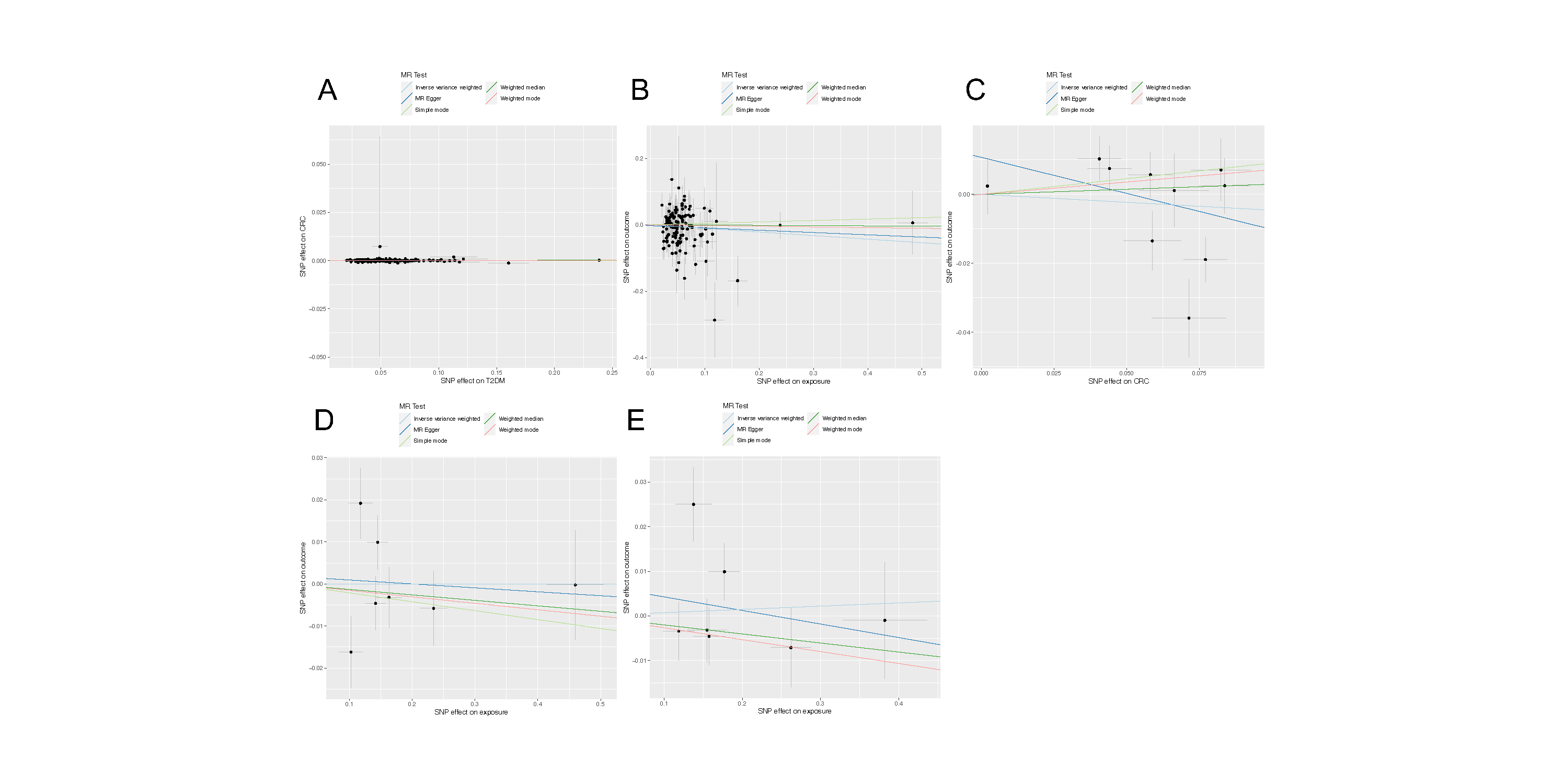

Supplement: Supplementary Figure 1 — Scatterplots of the causality between T2DM and CRC/IBD. [file Image_1.png]

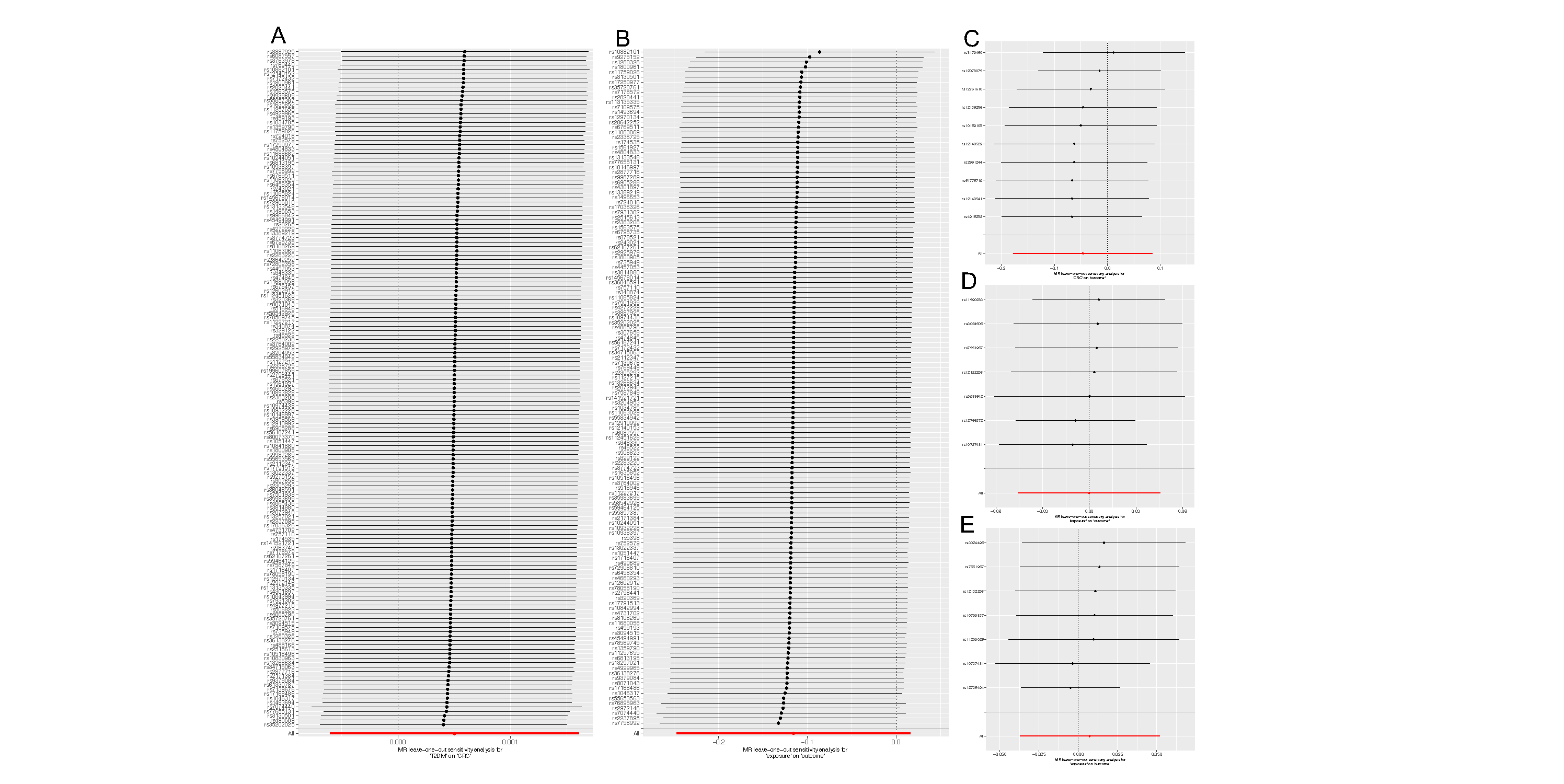

Supplement: Supplementary Figure 2 — Forest plots of the results of leave-one-out. [file Image_2.png]
